# Supplementary material for: Impact of Multidrug-Resistant Organisms on Severe Acquired Brain Injury Rehabilitation: An Observational Study
Source: Microorganisms. 2024 Apr 19;12(4):830. doi: 10.3390/microorganisms12040830 (PMC11052286; doi:10.3390/microorganisms12040830)
Supplement: Supplementary file 1 [file microorganisms-12-00830-s001.zip › microorganisms-2964872-supplementary.pdf]

Supplementary Materials

Supplementary Table S1. Factors associated with HAI incidence: results of univariate and age-adjusted Poisson regressions.

	Crude IRR[1] (95%CI)	p-value	Age adjusted IRR[1] (95%CI)	p-value
**Male**	1.31 (0.85 – 2.01)	0.221		
**Age**	1.03 (1.01 – 1.04)	<0.001		
**Aetiology (ref. traumatic)**		0.269		
**Vascular**	1.49 (0.89 – 2.49)			
**Other**	1.33 (0.84 – 2.08)			
**Comorbidity**	1.71 (1.11 – 2.65)	0.016	1.02 (0.61 – 1.70)	0.947
**LCF upon admission**	1.00 (0.88 – 1.14)	0.988		
**DRS upon admission**	1.01 (0.98 – 1.06)	0.475		
**CPE colonization (ref. No)**		0.009		0.116
**Upon admission**	1.55 (0.88 – 2.73)		1.55 (0.88 – 2.73)	
**During hospitalization**	2.24 (1.20 – 3.84)		1.76 (1.00 – 3.08)	
**Pre-admission surgery**	0.69 (0.46 – 1.02)	0.066		
**Surgery over hospitalization**	0.72 (0.48 – 1.06)	0.092		
**Number of transfers**	0.85 (0.67 – 1.06)	0.152		
**Number of Indwelling Catheter**	0.98 (0.84 – 1.13)	0.752		
**Indwelling Catheter days (ref. 0-14 days)**		0.012		0.154
**15-60 days**	1.53 (0.65 – 3.57)		1.51 (0.64 – 3.53)	
**>60 days**	2.45 (1.13 – 5.33)		1.96 (0.89 – 4.31)	
**Number of Central Venous Catheter**	1.09 (0.84 – 1.41)	0.541		
**CVC days (ref. 0)**		0.021		0.455
**1-31 days**	0.68 (0.39 – 1.17)		0.73 (0.42 – 1.25)	
**>31 days**	1.37 (0.88 – 2.14)		0.99 (0.61 – 1.61)	
**Tracheotomy tube (ref. No)**	2.01 (0.74 – 5.48)	0.170		
**Tracheotomy tube days (ref. 0-30 days)**		0.005		0.157
**31-120 days**	1.51 (0.69 – 3.28)		1.34 (0.62 – 2.92)	
**>120 days**	2.49 (1.24 – 4.98)		1.83 (0.89 – 3.77)	
**PEG or PEJ (ref. No)**	1.93 (1.12 – 3.34)	0.019	1.60 (0.91 – 2.80)	0.086
**PEG or PEJ days (ref. 0)**		0.010		0.142
**1-200 days**	2.52 (1.35 – 4.67)		1.88 (0.99 – 3.58)	
**>200 days**	1.72 (0.97 – 3.03)		1.50 (0.84 – 2.67)	

1. IRR: incidence rate ratio.

Legend: MDRO Multidrug Resistant Organisms, HAI healthcare associated infection, LCF levels of cognitive functioning, DRS disability rating scale, CPE carbapenemase-producing Enterobacteriaceae, PEG percutaneous endoscopic gastrostomy, PEJ percutaneous endoscopic jejunostomy.

Supplementary Table S2. Factors associated with MDRO HAI incidence: results of univariate and age-adjusted Poisson regressions.

	Crude IRR[1] (95%CI)	p-value	Age adjusted IRR[1] (95%CI)	p-value
**Male**	1.25 (0.55 – 2.86)	0.594		
**Age**	1.06 (1.03 – 1.09)	**<0.001**		
**Aetiology (ref.**		**0.003**		0.086
**traumatic)**	1.93 (0.65 – 5.76)		0.68 (0.19 – 2.48)	
**Vascular**	5.25 (1.87 – 14.7)		1.76 (0.49 – 6.29)	
**Other**				
**Comorbidity**	1.56 (0.66 – 3.69)	0.310		
**LCF upon admission**	1.20 (0.94 – 1.54)	0.134		
**DRS upon admission**	0.98 (0.91 – 1.05)	0.542		
**CPE colonization**		**0.019**		0.065
**(ref. No)**	4.77 (1.07 – 21.3)		4.59 (1.02 – 20.5)	
**Upon admission**	5.45 (1.23 – 24.2)		3.09 (0.68 – 14.1)	
**During hospitalization**				
**Pre-admission Surgery**	0.59 (0.28 – 1.26)	0.171		
**Surgery duruing hospitalization**	0.56 (0.26 – 1.20)	0.135		
**Number of transfers**	0.72 (0.45 – 1.14)	0.157		
**Indwelling catheter**	1.08 (0.82 – 1.41)	0.580		
**days (ref. 0-14days)**		0.207		
**15-60days**	1.21 (0.24 – 6.26)			
**>60 days**	2.39 (0.56 – 10.2)			
**Central venous catheter**	1.43 (0.86 – 2.37)	0.168		
**days (ref. 0)**		**<0.001**		**0.029**
**1-31 days**	0.10 (0.01 – 0.86)		0.13 (0.02 – 0.98)	
**>31 days**	1.62 (0.74 – 3.57)		0.85 (0.34 – 2.09)	
**Tracheostomy tube (ref. No)**	1.04 (0.25 – 4.38)	0.959		
**days (ref. 0-30days)**		0.094		
**31-120 days**	1.54 (0.30 – 7.95)			
**>120 days**	3.20 (0.75 – 13.7)			
**PEG or PEJ (ref. No)**	1.94 (0.67 – 5.60)	0.223		
**PEG or PEJ days**		0.096		
**(ref. 0)**	3.14 (0.99 – 10.0)			
**1-200 days**	1.49 (0.49 – 4.58)			
**>200 days**				

1. IRR: incidence rate ratio.

Legend: MDRO Multidrug Resistant Organisms, HAI healthcare associated infection, LCF levels of cognitive functioning, DRS disability rating scale, CPE carbapenemase-producing Enterobacteriaceae, PEG percutaneous endoscopic gastrostomy, PEJ percutaneous endoscopic jejunostomy.
